# Interpreting wide-angle X-ray scattering data using DNA duplexes as model systems

**DOI:** 10.1107/S2052252526007700

**Published:** 2026-09-01

**Authors:** Qingyue Hu, Vera Amirbekian, Sarah Uttormark, Hugh Robert Higinbotham, Lois Pollack

**Affiliations:** a School of Applied and Engineering Physics Cornell University Ithaca NY USA; UCL, United Kingdom

**Keywords:** wide-angle X-ray scattering, DNA duplexes, pair-distance distribution functions, base stacking, nucleic acid modeling

## Abstract

Small-angle X-ray scattering (SAXS) provides important information about global biomolecular solution structures yet is resolution limited. Extension to higher scattering angles through wide-angle X-ray scattering (WAXS) extends the resolution of solution measurements to single ångströms; however, interpretation of WAXS profiles is challenging. Here we establish a practical framework for interpreting WAXS measurements in terms of repeated real-space distances and specific structural motifs in nucleic acids. We compare experimentally measured scattering profiles from two 25-base-pair DNA duplexes, an AT-rich construct and a mixed-sequence duplex. Their SAXS profiles are similar but they exhibit strikingly different WAXS signatures. By systematically varying the reciprocal-space cutoff prior to indirect Fourier transforms, we show how increasing information content sharpens real-space resolution and reveals periodic features associated with base stacking and duplex geometry. Real-space distance distributions identify the dominant atomic contributors to these features and directly connect them to backbone separations, groove widths, and long-range stacking periodicities that vary in a sequence-dependent manner. Together, these results establish WAXS as a powerful tool for probing nucleic acid architecture and, in conjunction with time-resolved measurements, dynamics, and demonstrate that WAXS measurements provide a robust and generalizable route to extracting high-resolution structural information from solution measurements.

## Abstract 2

Wide-angle X-ray scattering (WAXS) profiles of DNA duplexes reveal peaks and troughs that reflect real-space periodic structural features. We draw on information-theory-limited Fourier transforms, atomistic modeling and residue-index correlation maps to link the observed WAXS peaks to known structural features of DNA duplexes, introducing a new way to characterize nucleic acid structures.

## 1. Introduction

High-resolution structural information plays a key role in our understanding of molecular biology and has supported many advances in biotechnology and medicine. Techniques capable of resolving ångström-scale structure such as X-ray crystallography and cryo-electron microscopy yield valuable insights but are restricted to static snapshots and require that samples are observed under non-physiological conditions (Saibil, 2022 ▸; Shi, 2014 ▸). Obtaining high-resolution structural measurements of macromolecules in solution, where conformational dynamics can be observed, remains a significant challenge.

Solution X-ray scattering provides an effective route to probing macromolecular structure under closer-to-physiological conditions. Small-angle X-ray scattering (SAXS) has been widely used to report global properties of macromolecules such as size, shape and oligomeric state, as well as to observe their conformational changes in solution (Chen & Pollack, 2016 ▸). Extension of measurements to higher scattering angle or momentum transfer, 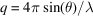 (with 2θ the scattering angle and λ the X-ray wavelength), the wide-angle X-ray scattering (WAXS) regime, increases sensitivity to internal structural features. In contrast to SAXS, WAXS scattering profiles often exhibit pronounced peaks and troughs. A growing body of work demonstrates that such high-*q* features encode detailed information about internal molecular organization in proteins, DNA and RNA (Minh & Makowski, 2013 ▸; Hong & Hao, 2009 ▸; Chen & Pollack, 2020 ▸; Chen *et al.*, 2022 ▸; Zielinski *et al.*, 2023 ▸; He *et al.*, 2021 ▸; Zuo *et al.*, 2006 ▸; Chamberlain *et al.*, 2023 ▸).

While it is straightforward to associate a scattering peak with a Bragg’s law equivalent length (*d* = 2π/*q*), solution scattering arises from randomly oriented molecules sampled over an ensemble of conformations and cannot be directly mapped to interatomic distances. Instead, peaks and troughs in solution-scattering profiles reflect the presence of repeated real-space distances visualized by the pairwise-distance distribution function *P*(*r*) (Warren, 1969 ▸; Moore, 1980 ▸). For molecules with regular internal organization, such as base stacking, helical geometry, and groove architecture in nucleic acids, these repeated length scales can give rise to especially prominent features in the WAXS signal (Köfinger & Hummer, 2013 ▸). As such, recent studies have demonstrated that WAXS features can be linked to specific structural parameters and even tracked in real time during RNA folding (Chen & Pollack, 2020 ▸; Chen *et al.*, 2022 ▸; Zielinski *et al.*, 2023 ▸). These studies benefit from the high flux available at X-ray sources, advances in detector technology, as well as the availability of computational models for comparison. WAXS has clear potential for resolving the structural dynamics of molecular ensembles but still lacks a clear methodology that connects high-*q* scattering features with underlying structural motifs in larger molecules, like nucleic acids. Here, using DNA duplexes as model systems, we present a practical workflow connecting real-space distance distributions derived from WAXS profiles with atomistic modeling and correlation maps, to relate WAXS measurements with structural motifs.

## 2. Results

### 2.1. WAXS discriminates between DNA duplexes by sequence

To illustrate the sensitivity of WAXS signals to molecular features, we introduce a framework for interpreting WAXS measurements, using two different DNA sequences for illustration. Because WAXS profiles reflect the existence of repeated length scales in the molecules, comparisons between real-space distance distributions [*P*(*r*)] and molecular models are used. We describe the acquisition of WAXS profiles, their conversion via indirect Fourier transform (IFT) into real-space distributions, and finally the interpretation of repeated length scales based on comparison with molecular models.

**Figure 1:**
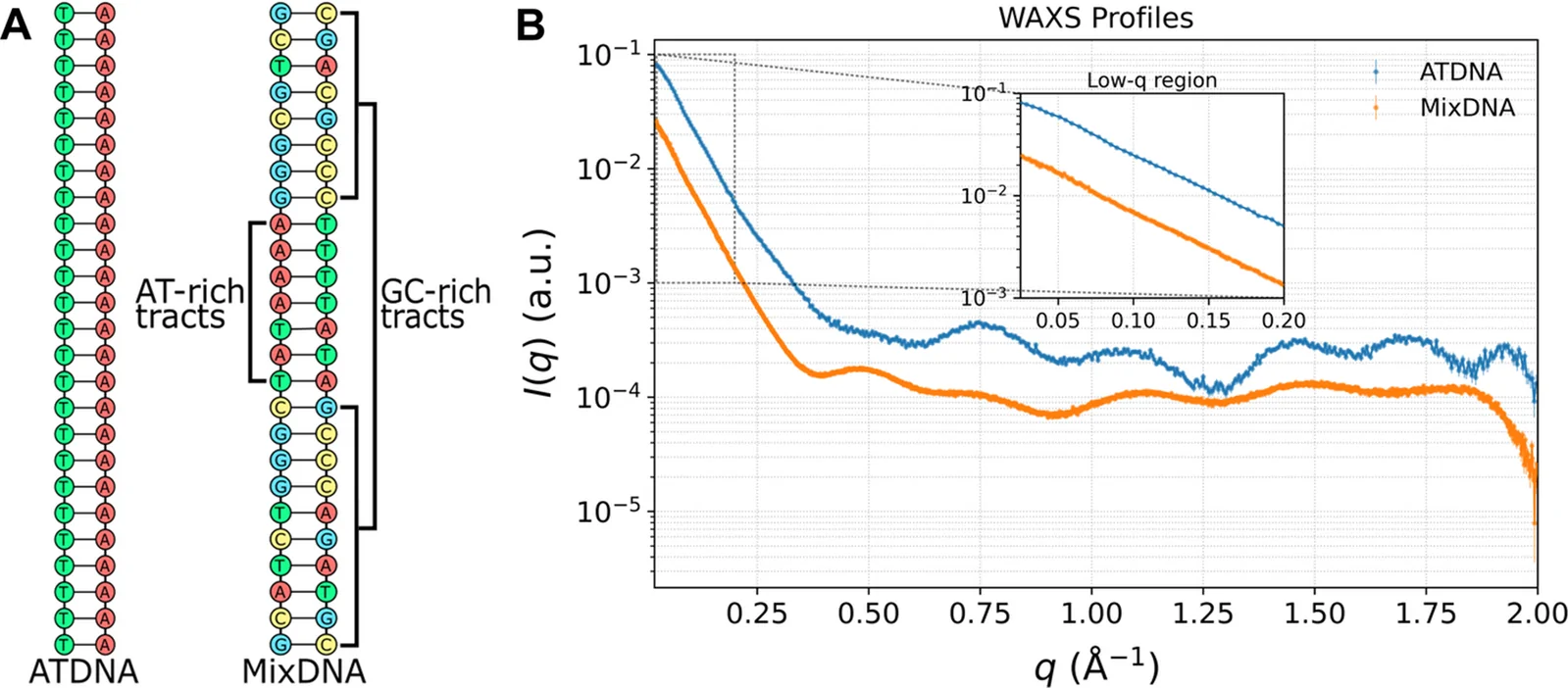
(*A*) Schematic drawing of the two 25-base-pair DNA duplexes studied: a pure AT duplex (‘AT DNA’, left) in which one strand contains only thymine bases and its complement only adenine, forming an A-tract-like B* variant, and a B-form mixed-sequence duplex (‘Mix DNA’, right) comprised of two 8–10-base-pair GC-rich segments at both ends flanking a 7-base-pair AT-rich core. (*B*) WAXS intensity profiles of AT DNA (blue) and Mix DNA (orange). The curves were offset for visual separation. The inset highlights the low-*q* (SAXS) region, where the shapes of the two curves are similar and yield similar *R*_g_ (21.9 ± 0.1 Å for AT DNA and 22.9 ± 0.1 Å for Mix DNA). At higher angles (*q* > 0.3 Å^−1^), WAXS profiles for AT DNA exhibit sharp periodic peaks between *q* values of 0.6–2.0 Å^−1^, whereas Mix DNA exhibits broader features in WAXS.

We first measured and compared the full solution-scattering profiles of two 25-base-pair DNA duplexes with different sequences, AT DNA and Mix DNA [Fig. 1 ▸(*A*)]. These molecules were previously characterized with solution scattering and molecular dynamics (MD) within a narrower *q* range (He *et al.*, 2021 ▸). DNA duplexes have sequence-dependent geometries, with mixed sequence molecules favoring the canonical B-form and AT-rich sequences favoring a variation with narrower groove widths (He *et al.*, 2021 ▸; Alexeev *et al.*, 1987 ▸) sometimes called B* (Zuo *et al.*, 2006 ▸; Kumar *et al.*, 2014 ▸). The Mix DNA construct used here contains two GC-rich tracts (8–10 base pairs each) at both ends and a central 7-base-pair AT-rich segment. Its bases are interleaved across strands rather than being confined to one, in contrast to AT DNA where all thymine nucleotides are on one strand and all adenine nucleotides are on the complementary strand.

Despite these compositional differences, the low-*q* SAXS regions (*q* < 0.15 Å^−1^) and Guinier-derived radii of gyration (*R*_g_) are similar for these two sequences, indicating comparable overall molecular dimensions [Fig. 1 ▸(*B*), inset; Fig. S1 of the supporting information]. A length series of AT-rich duplexes shows progressive emergence of the same duplex-associated WAXS peak pattern, supporting assignment of these oscillations to coherent duplex structure (Fig. S2). At higher scattering angles, the curves become distinct. AT DNA displays sharp, well-defined oscillations at *q* ≃ 0.6–2.0 Å^−1^, whereas Mix DNA shows broader and generally less pronounced peaks at higher *q*. These differences point to subtle but significant variations in internal duplex structure and illustrate the sensitivity of WAXS to sequence-dependent structural features that are difficult to quantify by SAXS alone. The relatively smooth appearance of the Mix DNA WAXS profile is primarily due to differences in experimental acquisition conditions between the two beamlines used. Protocols for extrapolating SAXS data to infinite dilution and assessing any contributions from interparticle interactions are illustrated in Fig. S1.

### 2.2. From reciprocal space to real space: *P*(*r*) reveals periodicity

**Figure 2:**
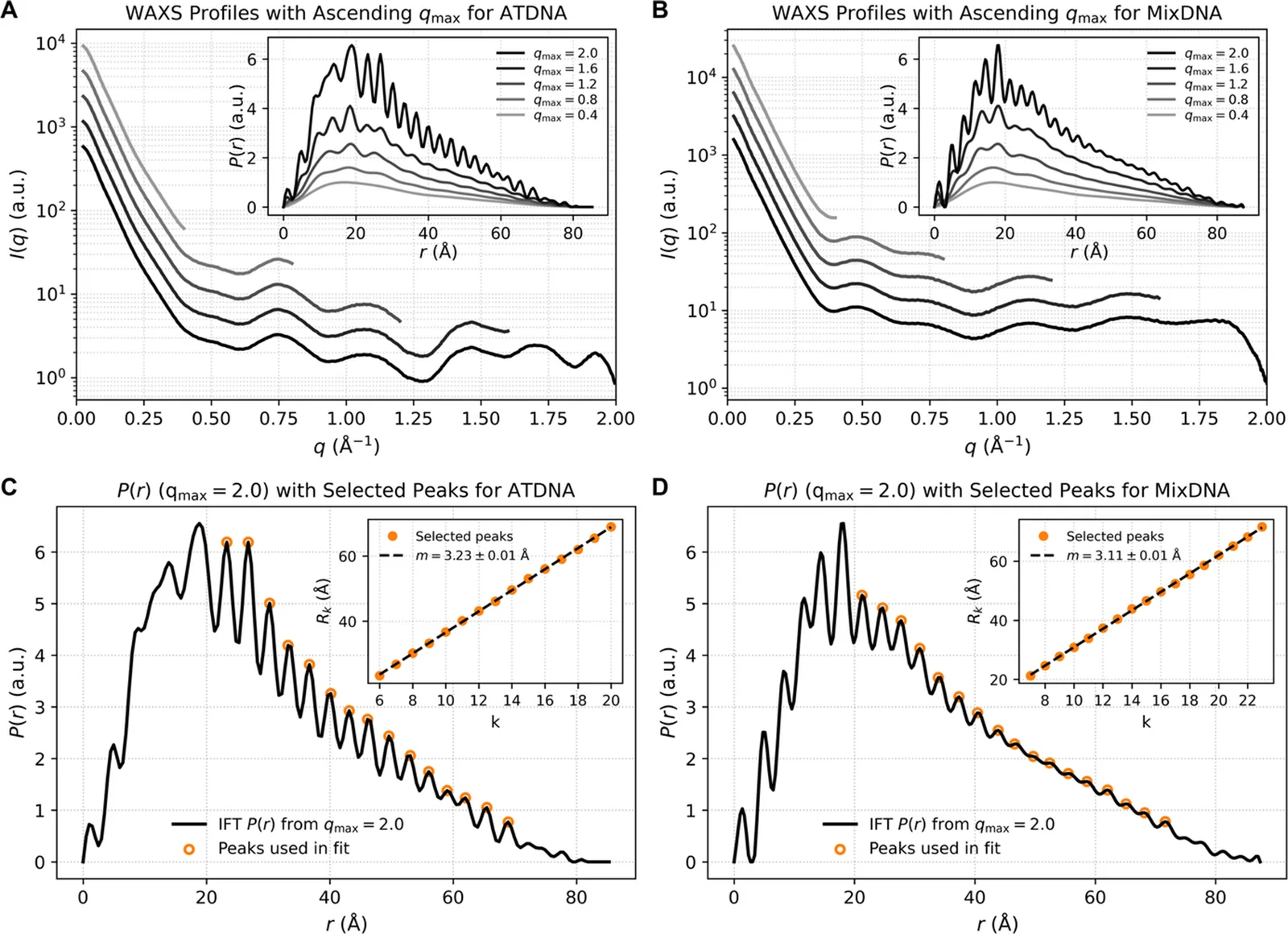
(*A*), (*B*) WAXS data of AT DNA and Mix DNA with increasing *q*_max_ cutoffs. Inset: *P*(*r*) distributions computed from AT DNA and Mix DNA WAXS data with progressive *q*_max_ cutoffs (0.4–2.0 Å^−1^, pale gray to black) using IFT. Higher *q*_max_ values reveal periodic peaks. (*C*), (*D*) Peak extraction from *P*(*r*) computed with *q*_max_ = 2.0 Å^−1^. Inset: peak position versus peak index (*k*) (between 20 and 70 Å) shows linear spacing with slope 3.23 ± 0.01 Å in AT DNA and 3.11 ± 0.01 Å in Mix DNA, demonstrating differences in base-stacking periodicity.

To interpret the features in the scattering profiles, we applied IFT of the experimental data to compute *P*(*r*), which reflects the distribution of electron–electron distances within the duplexes. We first illustrate that the resolution (distance between points) in the real-space curves is directly related to the upper *q* cutoff (*q*_max_) used in the transform. Following the information-theory framework of Moore and later developments by Rambo & Tainer (Moore, 1980 ▸; Rambo & Tainer, 2013 ▸, 2025 ▸), increasing *q*_max_ sharpens the real-space resolution (Δ*r* = π/*q*_max_) and increases the number of independent Shannon channels (*m* = *q*_max_*D*_max_/π, Table S2 of the supporting information). Consistent with this expectation, the smallest cutoff (*q*_max_ = 0.4 Å^−1^) yields a smooth bell-shaped *P*(*r*) dominated by the overall molecular dimensions [Figs. 2 ▸(*A*) and 2 ▸(*B*)]. As the highest *q* data used to perform the IFT increase to 0.8–1.2 Å^−1^, covering the first two scattering peaks of AT DNA and the first three of Mix DNA, distinct peaks emerge in the corresponding *P*(*r*) curves, at ∼12, 19 and 25 Å for both DNA duplexes. The *P*(*r*) shapes and peak positions are robust to different reconstruction methods, as shown by comparison of *GNOM* with Shannon pseudo-inverse and *DENSS* (Grant, 2022 ▸) reconstructions (Figs. S3 and S4).

At higher cutoffs (*q*_max_ = 1.6 Å^−1^) for AT DNA, additional fine structure appears on the right shoulder of *P*(*r*), and the ∼19 Å peak becomes more pronounced. Further inclusion of the scattering feature at 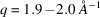 results in a series of regularly spaced peaks in the IFTs, more distinct in AT DNA than in Mix DNA. For Mix DNA, a weak but visually resolved shoulder is included as part of the indexed peak series. Peak indexing and linear regression reveal apparent periodic spacings of 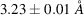 for AT DNA and 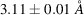 for Mix DNA. These spacings fall within the real-space length scale associated with base-stacking-related WAXS features, although both are somewhat shorter than the canonical ∼3.4 Å base-stacking repeat of B-form DNA. The reduced periodic increments suggest subtle deviations from canonical B-form stacking geometry, potentially reflecting sequence-dependent compaction or overlapping intramolecular correlations in the high-*q* region. In AT DNA, the shorter spacing is consistent with a B*-like structure, which has been associated with a narrower minor groove and tighter helical geometry (Kumar *et al.*, 2014 ▸). Association of the scattering feature near 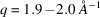 with base-stacking-related correlations has also been noted in WAXS studies of DNA duplexes through computation of WAXS profiles from simulated structures with artificially removed backbone atoms (Zuo *et al.*, 2006 ▸).

### 2.3. Atomistic models link scattering features to specific structural motifs

**Figure 3:**
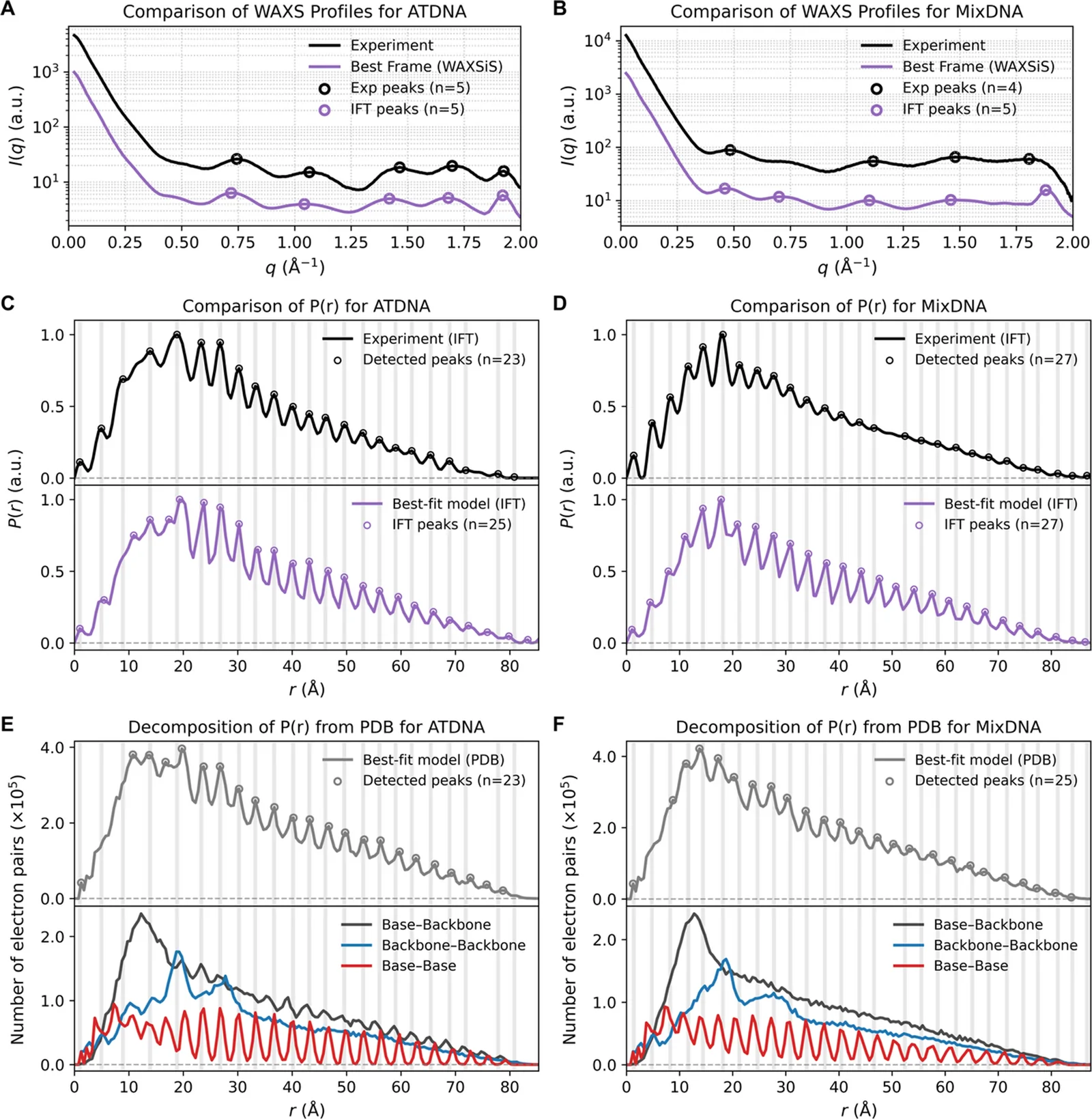
(*A*), (*B*) Comparison of experimental WAXS profiles displayed with a rolling average of nine points (black) with WAXS curves calculated from individual atomistic DNA models (purple). In both cases, we identified single models that reproduce the experimental scattering features. The WAXS profile computed from a single frame from AT DNA simulations shows near-perfect alignment of all five major peaks up to *q* = 2.0 Å^−1^. For the other sequence, a single frame was selected whose computed profile contains four peaks including a subtle shoulder near *q* ≃ 0.7 Å^−1^. (*C*), (*D*) Comparison of *P*(*r*) functions obtained by IFT of the experimental WAXS profiles (black) with IFT of *WAXSiS*-calculated scattering curves from the selected atomistic models (purple). Vertical gray bands denote prominent peaks in the experimental *P*(*r*) to facilitate comparison. (*E*), (*F*) Decomposition of the model-derived *P*(*r*) (gray) into base–base (red), base–backbone (black) and backbone–backbone (blue) atom-pair contributions, weighted by the number of electrons to approximate X-ray scattering intensity.

To assign specific *P*(*r*) peaks to real-space features of the molecules, we turned to atomistic DNA duplex models generated in a previous WAXS-driven MD study of these molecules (He *et al.*, 2021 ▸). This previous study only considered *q*_max_ values of 1.25 Å^−1^ and ensembles of structures were generated to match each profile. We computed the WAXS profile out to a *q*_max_ of 2.0 Å^−1^ for each structure in the theoretical ensemble. Scattering profiles were calculated with *WAXSiS*, which simulates scattering directly from the MD models including explicit solvent from all-atom MD trajectories (Chen & Hub, 2014 ▸; Knight & Hub, 2015 ▸). We selected the model structure for each duplex sequence whose scattering profile most closely matched our high-resolution data, using a χ^2^ metric to assess similarity [Figs. 3 ▸(*A*) and 3 ▸(*B*)]. We then computed the IFT of this best-frame *WAXSiS* curve using the same protocol as for the experimental scattering profiles [Figs. 3 ▸(*C*) and 3 ▸(*D*)]. The measured and computed *P*(*r*) curves agree well at distances above about 20 Å, but not perfectly below; the experimental *P*(*r*) for AT DNA shows a broad feature near 15–20 Å, whereas the model-derived curve resolves a subtle twin peak in the same region. When the experimental transform is extended to include higher *q* values, *q*_max_ = 2.03 Å^−1^ or above, the agreement improves and the peaks align well with those in the model-derived *P*(*r*) (Fig. S5). An ensemble *P*(*r*) comparison is provided in Fig. S6. At this high resolution, the remaining differences between experimental and model-derived profiles likely reflect structural heterogeneity in solution structures and the limitation of comparing an ensemble-averaged experimental profile with a single representative MD snapshot.

For Mix DNA, the simulated scattering profile correctly recapitulates the locations of the first four peaks but does not accurately capture the widest-angle broad peak measured in the experiment [Fig. 3 ▸(*B*)]. This discrepancy suggests that Mix DNA in solution has some variation relative to the representative structure selected. Accordingly, some disruption in periodicity is detected around 50 Å in the experimental *P*(*r*) relative to the model prediction.

With the atomistic models of our DNA duplexes, we can also compute *P*(*r*) directly from the atomic coordinates weighted by the number of electrons for a given atom type [Figs. 3 ▸(*E*) and 3 ▸(*F*)]. We used the Protein Data Bank (PDB) file to compute the distance between all pairs of atoms and separated the resulting histograms into three distinct groups representing: (i) base–base (red), (ii) backbone–backbone (blue) and (iii) base–backbone (black) atom pairs. For each category, we constructed a real-space distance histogram weighted by the product of atomic numbers (*Z*_1_ × *Z*_2_) of the involved atoms, thereby approximating the electron pair-distance distribution function. The weighted decompositions for both DNA constructs reveal that sharp regularly spaced peaks beyond ∼20 Å arise predominantly from interactions associated with stacked nucleobases, while the broad feature near 15–20 Å originates mainly from backbone–backbone distances. For AT DNA, the dominant features arise from backbone–base interactions (near 15 Å) and backbone–backbone interactions at 19 and 27 Å. These contacts dominate the *P*(*r*) curves, resulting in an asymmetric bell shape visible in all curves in Fig. 2 ▸(*A*). Peaks below about 30 Å appear and become distinct as higher *q* values are sampled. Once *q*_max_ exceeds 2.0 Å^−1^, a series of sharp regularly spaced peaks becomes visible at distances that exceed ∼20 Å. The corresponding spacings of 3.23 Å for AT DNA recurs here. Similar trends are observed in the Mix DNA sample, notably the recurrence of repeated peaks at 3.11 Å spacing. Interestingly, the peak near 52–54 Å appears sharper in AT DNA and more smeared in Mix DNA, suggesting differences in long-range organization.

### 2.4. Structural interpretation of peaks via correlation maps

**Figure 4:**
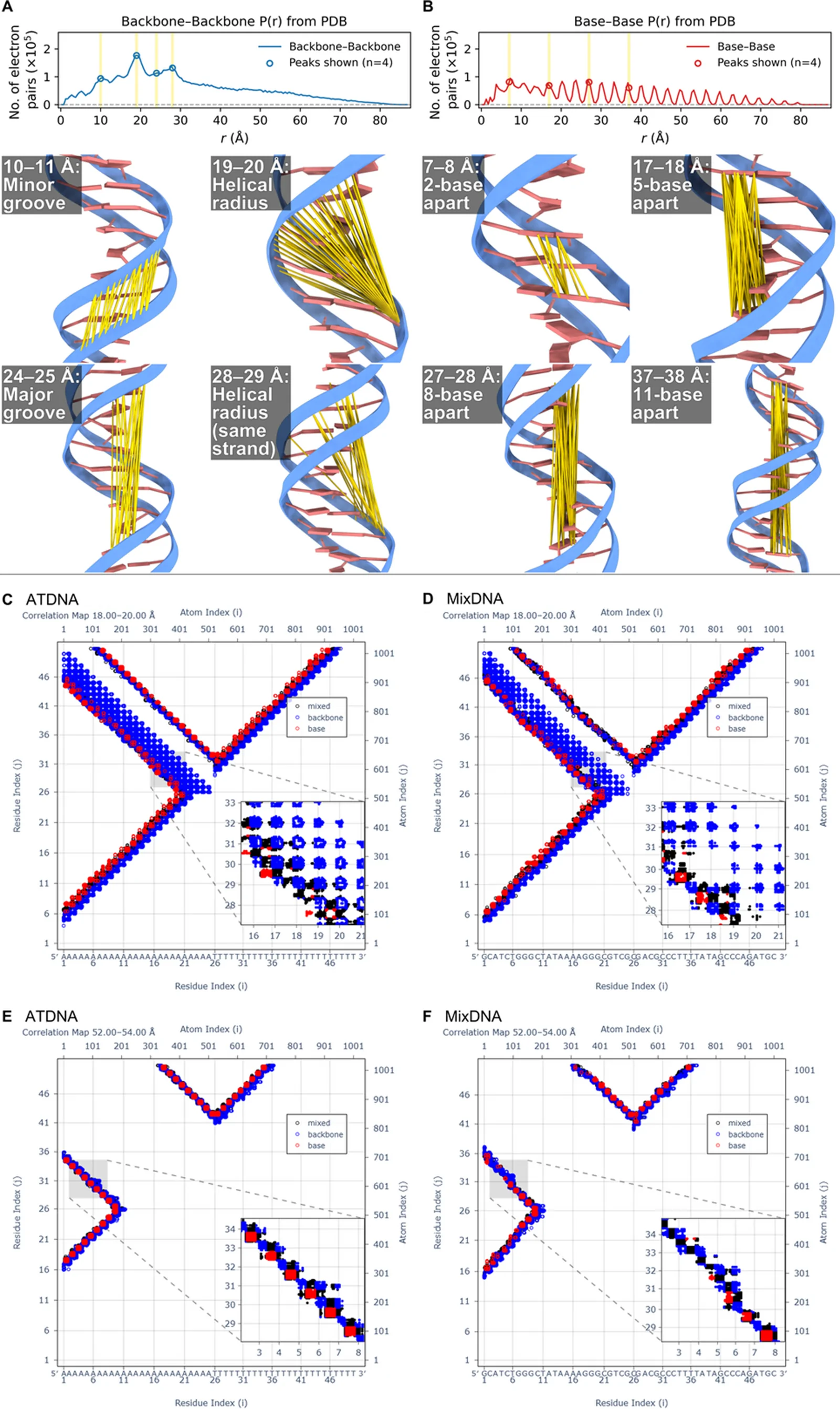
(*A*) *P*(*r*) derived for atoms only on the backbone (backbone–backbone pairs) for AT DNA with selected peaks highlighted (10–11, 19–20, 24–25 and 28–29 Å). Representative distances are visualized in atomic models using *ChimeraX*, illustrating separations spanning the minor groove, the cross-duplex helical radius, the major groove and the same-strand helical radius, respectively. (*B*) *P*(*r*) derived for atoms only associated with the base (base–base pairs) for AT DNA, highlighting peaks at 7–8, 17–18, 27–28 and 37–38 Å, corresponding to bases separated by 2, 5, 8 and 11 nucleotide steps along the duplex, respectively. (*C*), (*D*) Residue-index correlation maps for AT DNA and Mix DNA at 18–20 Å, showing highly regular ladders of backbone–backbone pairs in AT DNA and distorted irregular patterns in Mix DNA indicative of increased backbone heterogeneity. (*E*), (*F*) Residue-index correlation maps for AT DNA and Mix DNA at 52–54 Å, revealing long-range base-stacking correlations in AT DNA that are broadened and disrupted in Mix DNA.

With key distances identified from the *P*(*r*) analysis and atomic decompositions in Figs. 3 ▸(*E*) and 3 ▸(*F*), we next mapped each dominant length scale to the repeated atomic contacts that generate it using a combination of residue-index correlation maps and targeted structural visualization (Fig. 4 ▸).

We first focus on backbone-derived features in AT DNA [Fig. 4 ▸(*A*)]. Several prominent peaks in the backbone-only *P*(*r*) correspond to familiar duplex geometries. A broad feature spanning ∼8–12 Å arises from backbone separations across the minor groove. For clarity we highlight the center of this peak (10–11 Å) and visualize representative distances in the structural model. The strongest peak at 19–20 Å reflects a dominant cross-duplex geometry in which a backbone atom on one strand forms similar-length separations to several consecutive backbone atoms on the complementary strand, producing a fan-like pattern of distances. This motif corresponds to the effective helical radius of the duplex and constitutes the primary backbone contribution to the measured WAXS signal. A further peak at 24–25 Å arises from backbone atoms on opposing strands spanning the major groove region. Canonical B-form DNA major groove widths are often reported in the range of ∼12–22 Å, depending on the geometric definition. The separations identified here correspond to specific three-dimensional interatomic distances and therefore reflect the full helical geometry rather than an idealized projected groove width. Finally, the 28–29 Å feature corresponds to same-strand backbone geometries in which backbone atoms separated along the helix form arc-like distances that follow the helical contour, yielding characteristic separations longer than the cross-strand helical radius. An additional backbone-derived peak at 14–15 Å arises primarily from backbone atoms separated by three nucleotides along a single strand.

To illustrate base-derived contributions to the AT DNA *P*(*r*), we highlight peaks at 7–8, 17–18, 27–28 and 37–38 Å that correspond to bases separated by 2, 5, 8 and 11 steps along the duplex, respectively, reflecting periodic stacking along the helical axis [Fig. 4 ▸(*B*)]. The shortest-range feature is slightly larger than integer multiples of the previously derived 3.23 Å base-pair rise because these distances connect specific atoms on tilted base planes rather than vertical projections between successive base pairs. At larger separations, the distances approach multiples of the axial rise as the atom–atom connections become increasingly aligned with the helical axis.

To assess how sequence heterogeneity perturbs these dominant motifs, we examined residue-index correlation maps for AT DNA and Mix DNA at representative backbone-derived length scales [Figs. 4 ▸(*C*) and 4 ▸(*D*)]. At 18–20 Å, AT DNA displays highly regular straight diagonal ladders of backbone–backbone contacts, reflecting the persistence of the cross-duplex helical-radius geometry along the entire duplex. In contrast, the corresponding maps for Mix DNA exhibit bent diagonals and intermittently missing clusters, indicating distortions in cross-strand backbone organization and increased structural heterogeneity.

At longer base–base separations near 52–54 Å, where AT DNA exhibits sharper peaks in the *P*(*r*) distribution [Figs. 3 ▸(*C*) and 3 ▸(*D*), black curves], the correlation maps reveal extended diagonal clusters corresponding to stacking coherence over ∼16-base-pair steps [Figs. 4 ▸(*E*) and 4 ▸(*F*)]. These long-range correlations are substantially broadened and irregular in Mix DNA, consistent with reduced axial order. This loss of regularity is consistent with the alternating sequence architecture of the mixed duplex. Transitions between these blocks introduce subtle variations in stacking geometry and helical parameters, reducing the persistence of periodic correlations over long distances. The ∼16-base-pair spacing coincides with the junction between the central AT-rich segment and flanking GC-rich regions, supporting this interpretation, while weaker smeared features near 26–28 Å that correspond to approximately eight-base-pair separations may arise from the same sequence-dependent heterogeneity.

Together, these analyses demonstrate that combining *P*(*r*) decomposition, structural visualization and residue-index correlation maps provides a physical interpretation of WAXS-derived length scales. Distances in the 10–30 Å range primarily report on cross-duplex backbone geometries, whereas long-range (30–80 Å) separations reflect base-stacking order over progressively larger extents of the helix. The differing persistence of these features between AT DNA and Mix DNA explains the sharper oscillations observed in the AT DNA *P*(*r*) curve and highlights how sequence composition modulates duplex coherence across multiple spatial regimes.

## 3. Discussion

Our results demonstrate that WAXS can provide significant insight into structural differences that are difficult to distinguish by SAXS. WAXS profiles of different DNA duplexes diverge in a sequence-dependent manner. Transformation into real space and detailed comparison with atomistic modeling confirm that the WAXS profiles encode periodic distances corresponding to base stacking and duplex geometry. Thus, WAXS provides complementary data to those acquired by SAXS alone.

For highly regular DNA duplexes such as AT DNA, which have uniform sequence composition along their length, WAXS profiles exhibit sharp, well-defined oscillations in both reciprocal and real space, reflecting persistent axial order along the helix. In contrast, sequence heterogeneity in Mix DNA leads to broader and less regular features, consistent with increased variability in local geometry and reduced coherence of stacking over long distances. Rather than simply distinguishing canonical structural classes, these observations emphasize that WAXS is sensitive to subtle sequence-dependent departures from idealized duplex order. Differences between AT-rich- and mixed-sequence constructs, whether arising from local groove geometry, stacking perturbations or junction effects, can be interrogated quantitatively through comparison with atomistic models. In this way, WAXS provides a framework for probing how sequence composition modulates duplex organization in solution, setting the stage for the more general structural decomposition and future extensions discussed below.

By combining experimental scattering profiles with atomic modeling, we were able to correlate specific length scales in the *P*(*r*) curves with structural motifs. Most notably, the repetition of 18–20 Å is derived from cross-duplex backbone–backbone distances consistent with the helical diameter, while periodically spaced peaks for length scales beyond 20 Å arise almost exclusively from base–base contacts spanning defined numbers of base pairs. Furthermore, the correlation-map approach provides a direct visualization of how particular groups of residues contribute to each *P*(*r*) peak. This structural decomposition bridges the gap between measured scattering profiles and their origins in macromolecular structure.

New techniques to measure high-resolution structural information of dynamic molecular ensembles in solution are needed for physiologically relevant and time-resolved structural biology studies. As a practical benchmark, prior time-resolved WAXS studies (Zielinski *et al.*, 2023 ▸) indicate that reproducible WAXS features with signal-to-noise ratios of the order of 2 can still be structurally informative when they occur at consistent *q* positions. The approach presented here demonstrates the utility of WAXS combined with detailed molecular modeling to measure ångström-scale features of macromolecules in an aqueous environment. WAXS studies of more complex RNA and DNA macromolecules, including triplexes, hairpins, and higher-order assemblies that contain conserved repeated length scales, have already begun to reveal higher-resolution features in solution scattering (Chen *et al.*, 2022 ▸). Looking forward, integrating high-resolution WAXS with time-resolved approaches and advanced modeling pipelines is a promising new approach for revealing structural dynamics underlying biomolecular processes.

## 4. Material and methods

### 4.1. DNA sample preparation

The 25-mer AT DNA duplex (5′-TTTTTTTTTTTTTTTTTTTTTTTTT and its complement) and Mix DNA duplex (5′-GCA TCT GGG CTA TAA AAG GGC GTC G and its complement) were purchased from Integrated DNA Technologies (IDT). Both constructs were prepared in monovalent-ion buffers of similar composition: AT DNA in 150 m*M* NaCl, 10 m*M* NaMOPS (pH 7) and 50 µ*M* EDTA, and Mix DNA in 100 m*M* KCl, 10 m*M* NaMOPS (pH 7) and 20 µ*M* EDTA. The samples were buffer-exchanged six times with their corresponding buffers using a 3 kDa MWCO spin column at 14 000*g* for 10 min at 4°C, then annealed at 90°C for 3 min and cooled to room temperature for 15 min prior to measurement.

### 4.2. SAXS and WAXS data collection

SAXS data of both AT DNA and Mix DNA were collected on a BioXolver with a Genix laboratory X-ray source (Xenocs) using the MAX-HFLUX setup. WAXS measurements of AT DNA were acquired at the 16-ID Life Science X-ray scattering beamline (LiX) at the National Synchrotron Light Source II (NSLS-II) of Brookhaven National Laboratory using a Pilatus 1M detector with 15.14 keV beam energy. For AT DNA WAXS measurements, 60 µL of sample was injected into a flow cell, and data were collected as 10 frames with 0.5 s exposure per frame, giving a total exposure time of 5 s. Sample flow was used to minimize radiation damage. The WAXS data of Mix DNA were collected on the 7A1 beamline at the Cornell High Energy Synchrotron Source (CHESS) using a Pilatus 4M detector with 11.32 keV beam energy. For Mix DNA, data were collected as 100 frames with 1 s exposure per frame. The sample was loaded into a capillary, and the sample plug was oscillated during exposure to avoid repeatedly exposing the same sample volume and to minimize radiation damage. The specific *q* ranges, sample concentrations and exposure conditions for each dataset are summarized in Table S1.

### 4.3. Data processing

For S/WAXS data, beam centering and detector calibration were performed in *BioXTAS RAW* (Hopkins, 2024 ▸; Hopkins *et al.*, 2017 ▸; Nielsen *et al.*, 2009 ▸) using a silver behenate standard. Detector masks were applied to exclude edges, detector frames, abnormally high-intensity pixels, or the missing panel in the WAXS detector used at the LiX beamline. The scattering intensities were normalized to the transmitted beam and radially averaged, followed by scaling the sample curve by a factor of <0.2% to align to the right side of the first water peak (at *q* ≃ 2.1 Å^−1^, based on the assumption that there is no sample scattering in this region) with the buffer curve before buffer subtraction.

SAXS data acquired at DNA duplex concentrations of 100, 200, 500 and 1000 µ*M* were extrapolated to zero concentration using *PRIMUS* in *ATSAS* (Manalastas-Cantos *et al.*, 2021 ▸; Franke *et al.*, 2025 ▸) to exclude effects arising from intermolecular interactions. The extrapolated SAXS data were re-binned to match the data spacing of the corresponding WAXS curves and subsequently stitched by aligning the first ten points in the overlapping region *q* = 0.19–0.44 Å^−1^. The stitching was made such that the SAXS curve retained its original shape below *q* = 0.19 Å^−1^ and the WAXS curve retained its shape above *q* = 0.19 Å^−1^, yielding a continuous scattering profile across the full *q* range.

### 4.4. Computing scattering profiles and *P*(*r*)

Theoretical scattering profiles were computed from structural models using the *WAXSiS* web server (Chen & Hub, 2014 ▸; Knight & Hub, 2015 ▸) with default parameters and a scattering range up to *q* = 2.0 Å^−1^. Experimental *P*(*r*) distributions were obtained from *I*(*q*) using *GNOM* [*ATSAS* (Manalastas-Cantos *et al.*, 2021 ▸; Svergun, 1992 ▸)] implemented in *BioXTAS RAW*, which performs an IFT under Tikhonov regularization (Tikhonov & Arsenin, 1977 ▸). Unless otherwise stated, *RAW* default settings were used without enforcing *P*(*r*) = 0 at *r* = *D*_max_.

For *P*(*r*) reconstruction, *D*_max_ was fixed using the maximum molecular dimension estimated from the corresponding atomistic PDB model: 85.3 Å for AT DNA and 87.4 Å for Mix DNA, consistent with data showing that these molecules are monomers under the conditions studied. To assess robustness with respect to our choice of parameters, small variations around these *D*_max_ values (±1–2 Å) were tested together with different values of the smoothing parameter ALPHA. Over *q* ε [0.01–2.00] Å^−1^ with *D*_max_ = 85.3 Å, we scanned ALPHA from 0.1α to 10α, where α is *GNOM*’s automatic choice. We selected a reasonable value that (i) naturally fitted the five dominant peaks in *I*(*q*), and (ii) kept *R*_g_ and *I*(0) consistent with the results from Guinier analysis. The *GNOM* regularization procedure, ALPHA-scan strategy, and criteria used to avoid reconstruction artefacts are described in the supporting information, Section S4.

Uncertainties in WAXS peak locations were estimated from the standard error of the corresponding linear regression. For *P*(*r*) peak positions, formal peak-position uncertainties are not directly reported by the reconstruction software; therefore, we estimated the uncertainty from the *r*-grid spacing used in the reconstruction. With a grid spacing of 0.5 Å, *P*(*r*) peak positions were reported with a conservative estimated uncertainty of ±1 Å.

## Supplementary Material

Supporting information. DOI: 10.1107/S2052252526007700/ro5049sup1.pdf

SASBDB reference: 25-base-pair AT DNA duplex, SASDYJ9

SASBDB reference: 25-base-pair Mix DNA duplex, SASDYK9

## Data Availability

SAXS and WAXS profiles are deposited in the SASBDB with accession codes: SASDYJ9 (AT DNA) and SASDYK9 (Mix DNA).

## References

[bb1] Alexeev, D. G., Lipanov, A. A. & Skuratovskii, I. Ya. (1987). *Nature* **325**, 821–823. 10.1038/325821a0 3821870

[bb2] Chamberlain, S. R., Moore, S. & Grant, T. D. (2023). *bioRxiv*, 2023.06.02.543451.

[bb3] Chen, P. & Hub, J. S. (2014). *Biophys. J.* **107**, 435–447. 10.1016/j.bpj.2014.06.006 PMC4104061 25028885

[bb4] Chen, Y. & Pollack, L. (2016). *WIREs RNA* **7**, 512–526. 10.1002/wrna.1349 PMC4909577 27071649

[bb5] Chen, Y.-L., He, W., Kirmizialtin, S. & Pollack, L. (2022). *Cell. Rep. Phys. Sci.* **3**, 100971. 10.1016/j.xcrp.2022.100971 PMC9351628 35936555

[bb6] Chen, Y.-L. & Pollack, L. (2020). *IUCrJ* **7**, 870–880. 10.1107/S2052252520008830 PMC7467162 32939279

[bb40] Franke, D., Gräwert, T. & Svergun, D. I. (2025). *J. Appl. Cryst.* **58**, 1027–1033. 10.1107/S1600576725002481 PMC12135997 40475945

[bb7] Grant, T. D. (2022). *J. Appl. Cryst.* **55**, 1116–1124. 10.1107/S1600576722006598 PMC9533761 36249494

[bb8] He, W., Chen, Y.-L., Pollack, L. & Kirmizialtin, S. (2021). *Sci. Adv.* **7**, eabf6106. 10.1126/sciadv.abf6106 PMC8064643 33893104

[bb9] Hong, X. & Hao, Q. (2009). *Appl. Phys. Lett.* **94**, 083903.

[bb10] Hopkins, J. B. (2024). *J. Appl. Cryst.* **57**, 194–208. 10.1107/S1600576723011019 PMC10840314 38322719

[bb11] Hopkins, J. B., Gillilan, R. E. & Skou, S. (2017). *J. Appl. Cryst.* **50**, 1545–1553. 10.1107/S1600576717011438 PMC5627684 29021737

[bb12] Knight, C. J. & Hub, J. S. (2015). *Nucleic Acids Res.* **43**, W225–W230. 10.1093/nar/gkv309 PMC4489308 25855813

[bb13] Köfinger, J. & Hummer, G. (2013). *Phys. Rev. E* **87**, 052712. 10.1103/PhysRevE.87.052712 23767571

[bb14] Kumar, S., Newby Spano, M. & Arya, D. P. (2014). *Biopolymers* **101**, 720–732. 10.1002/bip.22448 PMC4112085 24281844

[bb15] Manalastas-Cantos, K., Konarev, P. V., Hajizadeh, N. R., Kikhney, A. G., Petoukhov, M. V., Molodenskiy, D. S., Panjkovich, A., Mertens, H. D. T., Gruzinov, A., Borges, C., Jeffries, C. M., Svergun, D. I. & Franke, D. (2021). *J. Appl. Cryst.* **54**, 343–355. 10.1107/S1600576720013412 PMC7941305 33833657

[bb16] Minh, D. L. & Makowski, L. (2013). *Biophys. J.* **104**, 873–883. 10.1016/j.bpj.2012.12.019 PMC3576546 23442966

[bb17] Moore, P. B. (1980). *J. Appl. Cryst.* **13**, 168–175.

[bb18] Nielsen, S. S., Toft, K. N., Snakenborg, D., Jeppesen, M. G., Jacobsen, J. K., Vestergaard, B., Kutter, J. P. & Arleth, L. (2009). *J. Appl. Cryst.* **42**, 959–964.

[bb19] Rambo, R. P. & Tainer, J. A. (2013). *Nature* **496**, 477–481. 10.1038/nature12070 PMC3714217 23619693

[bb20] Rambo, R. P. & Tainer, J. A. (2025). *Biophys. J.* **124**, 2511–2522. 10.1016/j.bpj.2025.06.031 PMC12414664 40581817

[bb21] Saibil, H. R. (2022). *Mol. Cell* **82**, 274–284. 10.1016/j.molcel.2021.12.016 35063096

[bb22] Shi, Y. (2014). *Cell* **159**, 995–1014. 10.1016/j.cell.2014.10.051 25416941

[bb23] Svergun, D. I. (1992). *J. Appl. Cryst.* **25**, 495–503.

[bb24] Tikhonov, A. N. & Arsenin, V. Y. (1977). *Solutions of Ill-posed Problems*. Washington, DC: Winston & Sons.

[bb34] Warren, B. E. (1969). *X-ray Diffraction*. Reading, MA: Addison-Wesley.

[bb35] Zielinski, K. A., Sui, S., Pabit, S. A., Rivera, D. A., Wang, T., Hu, Q., Kashipathy, M. M., Lisova, S., Schaffer, C. B., Mariani, V., Hunter, M. S., Kupitz, C., Moss, F. R., Poitevin, F. P., Grant, T. D. & Pollack, L. (2023). *Sci. Adv.* **9**, eadj3509. 10.1126/sciadv.adj3509 PMC10530093 37756398

[bb36] Zuo, X., Cui, G., Merz, K. M., Zhang, L., Lewis, F. D. & Tiede, D. M. (2006). *Proc. Natl Acad. Sci. USA* **103**, 3534–3539. 10.1073/pnas.0600022103 PMC1383498 16505363

