# Supplementary material for: Interpreting wide-angle X-ray scattering data using DNA duplexes as model systems
Source: IUCrJ. 2026 Sep 1;13(Pt 5):650–8. doi: 10.1107/S2052252526007700 (PMC13548833; doi:10.1107/S2052252526007700)
Supplement: Supplementary file 1 [file m-13-00650-sup1.pdf]

IUCrJ

Volume 13 (2026)

Supporting information for article:

Interpreting wide-angle X-ray scattering data using DNA duplexes as model systems

Qingyue Hu, Vera Amirbekian, Sarah Uttormark, Hugh Robert Higinbotham and Lois Pollack

S1. Low-q behavior and Guinier analysis

For AT DNA, there is no clear evidence for concentration-dependent association. The 100 and 200 µM Guinier plots are mostly linear and yield R_g values of ~21–22 Å, consistent with monomeric duplex dimensions. At the WAXS concentration (500 µM), the apparent R_g decreases to 18.73 Å, and it decreases further to 17.06 Å at 800 µM; this trend is consistent with weak repulsive interparticle effects at higher concentration.

For Mix DNA, the slight low-q upturn occurs only at the lowest DNA concentrations and is likely a result of background subtraction errors when the scattering signal is small. We note that this feature is confined to the lowest-q region and does not affect the high-q WAXS features used to assign groove/base-stacking distances. We therefore retain the current SWAXS processing pipeline while explicitly documenting the low-q behavior and Guinier fitting range.

When present, the structure-factor effects are limited to the lowest-q region and are far from the SWAXS merge region. Therefore, these effects do not affect the WAXS-dominated real-space features used for structural interpretation. For completeness, Figure S1 shows how the Guinier parameters vary with fitting range.

During SWAXS stitching, WAXS intensities and their associated uncertainties were multiplied by the same scale factor used to align the WAXS curve to the SAXS overlap region, while SAXS uncertainties were retained from the processed SAXS data. The different q-point densities for the two constructs reflect differences in X-ray source, detector configuration, detector pixel sampling, and q-sampling. The stitched SWAXS files retain propagated errors. The final stitched file uses SAXS points below the seam and scaled WAXS points above the seam; in overlapping q regions, WAXS points are retained and near-duplicate SAXS points are removed.

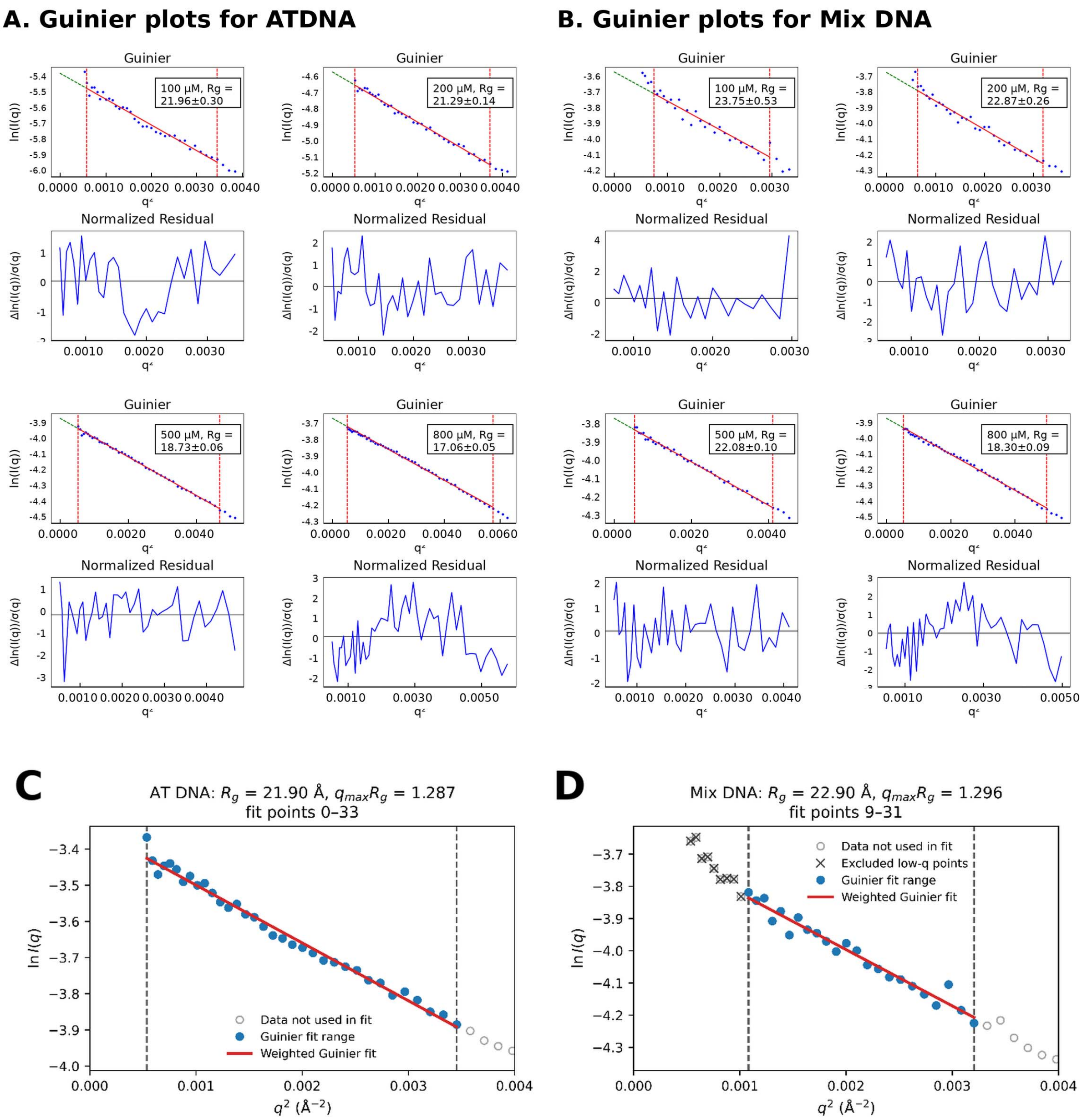

E

Guinier fit-range sensitivity

Sample	First point	Last point	n	q_{min}	q_{max}	R_g (Å)	$q_{max}R_g$
AT DNA	0	33	34	0.0232	0.0588	21.900	1.287
AT DNA	1	34	34	0.0243	0.0598	21.718	1.300
AT DNA	2	34	33	0.0253	0.0598	21.701	1.299
AT DNA	3	33	31	0.0264	0.0588	21.801	1.281
AT DNA	4	33	30	0.0275	0.0588	21.773	1.280
Mix DNA	8	31	24	0.0318	0.0566	22.857	1.294
Mix DNA	9	31	23	0.0329	0.0566	22.901	1.296
Mix DNA	10	31	22	0.0340	0.0566	22.776	1.289
Mix DNA	11	31	21	0.0350	0.0566	22.714	1.286
Mix DNA	12	32	21	0.0361	0.0577	22.523	1.299

Figure S1 Guinier analysis and low-q fit-range sensitivity for AT DNA and Mix DNA. Individual Guinier plots for AT DNA (A) and Mix DNA (B) at 100, 200, 500, and 800 µM. (C, D) Zero-concentration extrapolated SAXS Guinier plots for AT DNA (C) and Mix DNA (D). (E) Fit-range sensitivity analysis. Blue points indicate data included in Guinier fits, grey points indicate nearby data

excluded from fitting, red lines show Guinier fits, and dashed vertical lines mark the fitted q ranges. For Mix DNA, the lowest-q points show a weak upturn, likely a result of background subtraction issues, and were excluded from the reported Guinier fit. The fitted R_g values remain stable across nearby valid fitting windows satisfying $q_{max}R_g$ < 1.3, supporting the reported dimensions and indicating that the conclusions are not sensitive to the Guinier fit range.

S2. Data-collection q-ranges and acquisition details

The usable q-ranges for SAXS, WAXS, and final stitched SWAXS profiles are summarized in Table S1.

Table S1 Data-collection q-ranges and acquisition details.

Dataset	Instrument / beamline	Concentration	Usable q-range	Final merged q-range	Exposure
AT DNA SAXS	BioXolver / Xenocs	100–800 µM	0.0232–0.4405 Å⁻¹	0.0232–2.0300 Å⁻¹	120 s × 10
Mix DNA SAXS	BioXolver / Xenocs	100–800 µM	0.0232–0.4405 Å⁻¹	0.0232–2.1010 Å⁻¹	120 s × 10
AT DNA WAXS	NSLS-II LiX 16-ID	500 µM	0.1834–2.0300 Å⁻¹	0.0232–2.0300 Å⁻¹	0.5 s × 10
Mix DNA WAXS	CHESS 7A1	500 µM	0.1831–2.1010 Å⁻¹	0.0232–2.1010 Å⁻¹	1 s × 100

S3. Length-dependent evolution of WAXS features in AT DNA duplexes

To examine how duplex length influences wide-angle scattering features, we compared WAXS profiles of AT-rich DNA duplexes spanning 6 to 25 base pairs. The shortest construct, AT DNA6, exhibits only a weak and broad feature near $q \approx 1.1$ Å⁻¹, with other characteristic duplex peaks largely absent, consistent with the expectation that a minimum of roughly one helical turn (~10 bp) is required to establish well-defined groove geometry and long-range stacking correlations in duplex DNA.

Upon increasing the duplex length to 12 base pairs, clear features emerge at $q \approx 0.75$, 1.1, and 1.9 Å⁻¹, although the peaks near 1.4 and 1.7 Å⁻¹ remain poorly defined. Further extension to 18 base pairs results in substantially sharper peaks at $q \approx 0.75$ and 1.7 Å⁻¹, indicating the development of longer-range duplex correlations. Notably, the base-stacking-associated peak near $q \approx 1.9$ Å⁻¹, while present in AT DNA18, is still less pronounced than in AT DNA25. Together, these trends demonstrate a progressive strengthening of DNA duplex WAXS signatures with increasing DNA length, consistent with enhanced structural coherence along the helical axis. Although these measurements were

collected using different monovalent-salt conditions (2 mM NaCl for AT DNA6/12/18 versus 150 mM NaCl for AT DNA25), duplex formation is robust over a wide range of monovalent ion concentrations, and the consistent, length-dependent appearance of peak positions and relative intensities indicates that the dominant trends arise from duplex length rather than buffer composition.

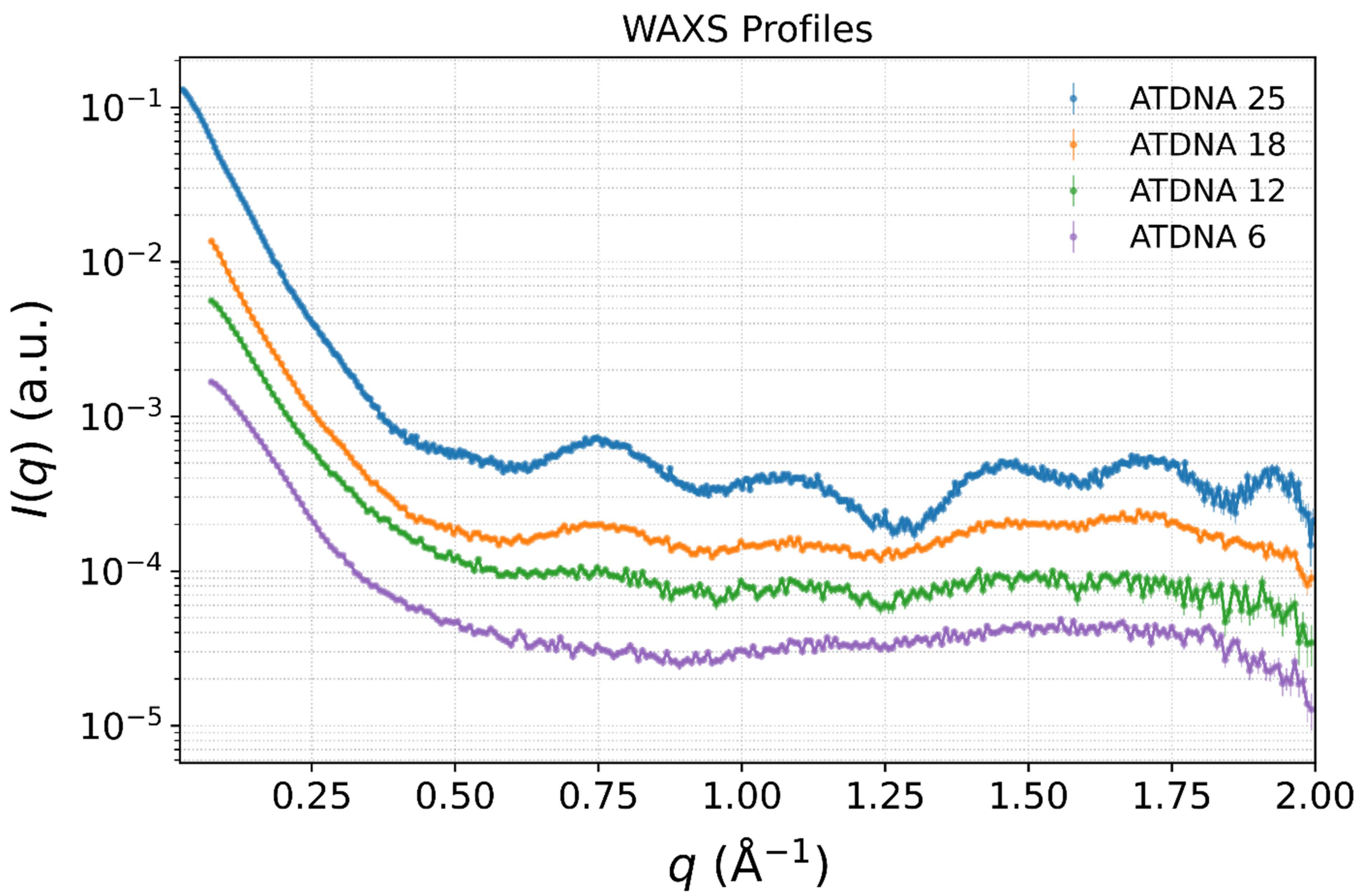

Figure S2 Wide-angle X-ray scattering (WAXS) profiles of AT-rich DNA duplexes with lengths of 6, 12, 18, and 25 base pairs are shown. AT DNA25 curve is identical to that shown in Figure 1 and was collected at a synchrotron under high-salt conditions (150 mM NaCl). The shorter constructs (AT DNA6, AT DNA12, and AT DNA18) were measured at 1000 µM DNA concentration using a laboratory-based X-ray source (Xenocs, MAX-HFLUX configuration) in a low-salt buffer (2 mM NaCl, 1 mM MOPS pH 7, 50 µM EDTA). For AT DNA6, the complementary 6A and 6T strands were connected via a short 5-cytosine linker to facilitate sample preparation and buffer exchange. Despite differences in experimental conditions, all constructs form duplex structures, enabling a qualitative comparison of length-dependent WAXS features. Prominent duplex-associated peaks near $q \approx 0.75$, 1.1, 1.4, 1.7, and 1.9 $Å^{-1}$ become increasingly pronounced with increasing duplex length.

S4. GNOM method for Indirect Fourier Transform

GNOM was used to compute pair-distance distribution functions $P(r)$ from the experimental scattering profiles using regularized indirect Fourier transform. The regularization parameter α controls the level of smoothing of the reconstructed distribution and is selected automatically in

GNOM by optimizing a composite perceptual score that incorporates criteria such as reciprocal-space agreement, smoothness, stability, and positivity. Additional details of this optimization procedure are described in the original GNOM documentation (Svergun, 1992).

To ensure that reconstruction artifacts did not influence our structural interpretation, we explicitly scanned α and verified that the positions of the major $P(r)$ peaks were insensitive to its value over a broad range. In cases where GNOM's automatic selection produced over-smoothed distributions at low q_{max}, we adopted the smallest α that preserved reproducible features in $I(q)$, yielded smooth but structured $P(r)$ curves, and avoided overlap of the Guinier region with the real-space distribution. These low-q_{max} reconstructions were used only to illustrate resolution effects and were not employed for structural interpretation.

S5. $P(r)$ robustness with respect to q_{max} and alternative transforms

To address concerns about oversampling and over-interpretation in real-space reconstructions, we complemented GNOM-based IFT with an information-theory-limited approach (Grant, 2022; Rambo & Tainer, 2025). These analyses evaluate whether the number of independent data points in the experimental q-range is sufficient to justify structural features in $P(r)$ and penalize reconstructions whose effective complexity exceeds the information content of the data.

Across all reconstructions we verified that (i) residuals were effectively uncorrelated, and (ii) GNOM, Shannon pseudo-inverse (SPI), and DENSS reconstructions produced mutually consistent $P(r)$ envelopes and peak positions over the same q-range. The full comparison of real-space reconstructions using the scoring algorithm from ScÅtter, including scans over q_{max}, D_{max}, and α, is provided in Figure S3 and Table S3 and supports our use of GNOM reconstructions at high q_{max} without evidence of overfitting.

For a fixed maximum particle dimension D_{max} = 85.3 Å for AT DNA, the number of independent Shannon channels m and the corresponding real-space resolution $\Delta r = \pi/q_{max}$) were calculated for each q_{max} value and are summarized in Table S2. These values quantify the smallest resolvable length scale in $P(r)$ and provide a conservative bound on the separability of nearby peaks. For example, resolving base-stacking distances near d ≈ 3.4 Å requires $q_{max} \gtrsim 0.95$ Å^{-1}, while robust separation of higher-order periodic features requires $q_{max} \geq 1.9$ Å^{-}.

To resolve a feature of width or spacing d as a distinct peak in $P(r)$ without aliasing, the transform should have about two resolution elements across it, i.e., $\Delta r \sim d/2$. For AT DNA with fixed D_{max} = 85.3 Å, Table S2 reports m and Δr for each q_{max}. For the base-stacking spacing d ≈ 3.4 Å, the detectability bound is ~0.95 Å^{-1}, while robust separation for the peaks in $P(r)$ should have $q_{max} \gtrsim$ 1.90 Å^{-1}.

Table S2 For a fixed maximum particle dimension (D_{max} = 85.3 Å), the number of independent Shannon channels (m) and the corresponding real-space resolution ($\Delta r = \pi/q_{max}$) are calculated for different q_{max} values.

Shannon sampling estimates of AT DNA scattering data

q_{max} (Å^{-1})	m (channels)	Δr (Å)
0.4	10.86	7.85
0.8	21.72	3.93
1.2	32.58	2.62
1.6	43.44	1.96
2.0	54.30	1.57

S6. PDB-derived $P(r)$ decomposition

In both Figure S5 and Figure 3C, D the IFT curve from the model was interpolated onto the same grid as both experimental curves to ensure one-to-one correspondence with the experimental sampling. The PDB-derived $P(r)$ was calculated from all pairwise atomic distances within the structure, with electron-count weighting ($Z_i Z_j$) applied to each atomic pair. The resulting distance histogram was binned uniformly with a bin width of 0.5 Å. Peaks were identified on each curve using the same detection criteria (minimum separation 1 Å, relative prominence 0.02 of the local maximum), allowing consistent comparison of structural features across experimental, reconstructed, and model-based representations. Across SPI, GNOM, and DENSS, standardized residuals were structureless (DW ≈ 1.8-1.9) and Psi penalties were near zero, indicating no evidence of oversampling. While SPI favored a slightly shorter effective D_{max} (~76-77 Å), GNOM with the physically justified D_{max} = 85.3 Å yielded comparable residual whiteness and stable $P(r)$ features, we therefore retained 85.3 Å for structural interpretation.

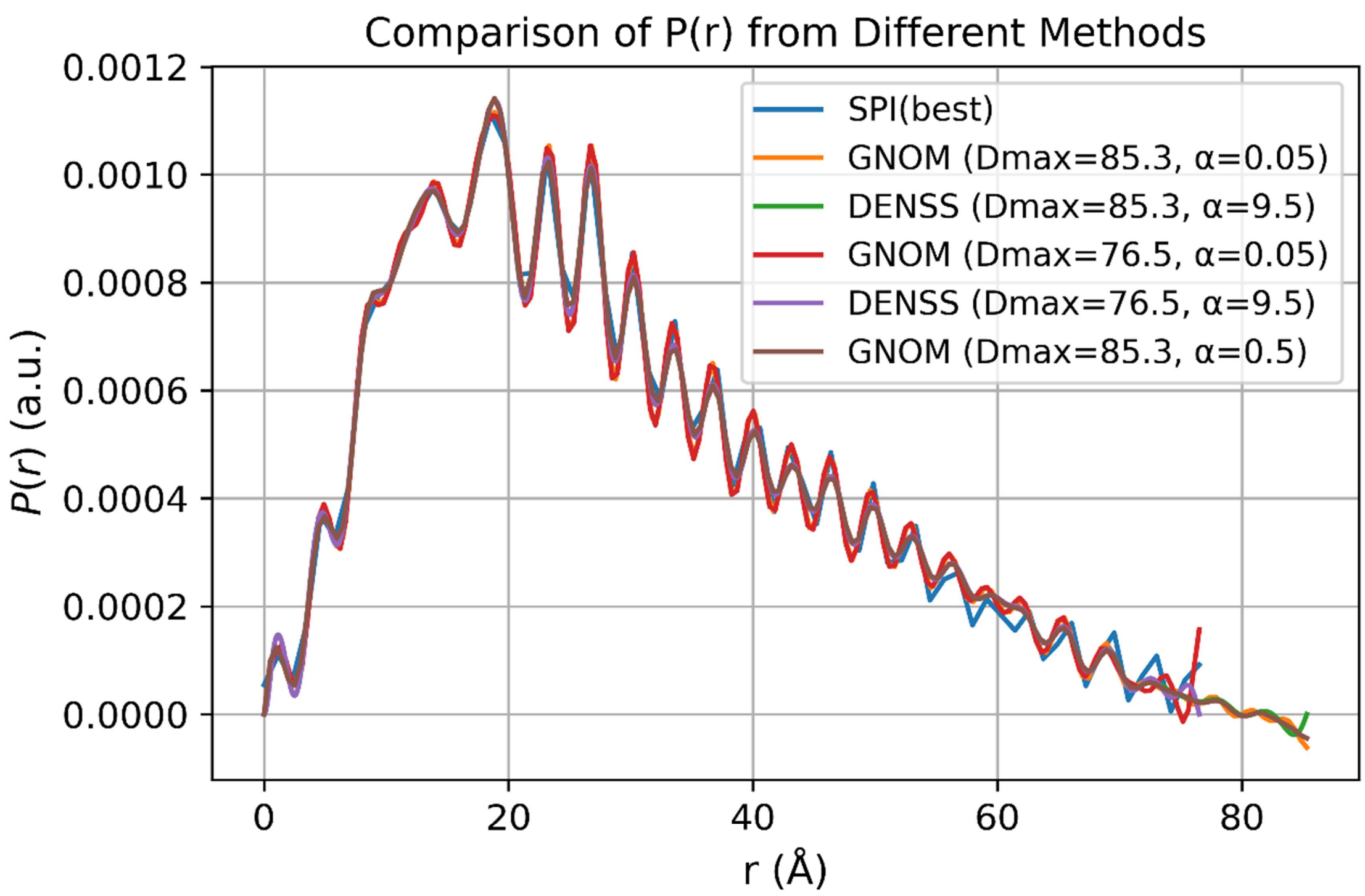

Figure S3 Comparison of $P(r)$ computed by (i) SPI (Shannon-limited pseudo-inverse; best D_{max} by overall Score), (ii) GNOM (indirect Fourier transform with Tikhonov regularization; D_{max} fixed at the duplex prior 85.3 Å; alphas as labeled), and (iii) DENSS (Grant, 2022). Curves were least-squares scaled to the SPI best $P(r)$ for visual comparison. All three methods agree on the envelope and principal features in 10–65 Å.

Table S3 Model-selection metrics across methods and D_{max}

D_{max}	Method	alpha	m	AIC	AICc	DW	Score
76.5	DENSS	9.5	48	728.90	740.23	1.78	74.89
85.3	DENSS	9.5	54	738.70	753.22	1.79	76.16
76.5	GNOM	0.05	48	722.29	733.62	1.81	74.13
85.3	GNOM	0.05	54	733.01	747.53	1.81	75.50
85.3	GNOM	0.5	54	740.26	754.78	1.79	76.33
76.5	SPI		48	695.48	706.82	1.91	71.03
76	SPI		48	695.66	707.00	1.91	71.05
77	SPI		48	696.19	707.53	1.91	71.11
75.5	SPI		48	696.31	707.65	1.91	71.12

77.5	SPI	49	697.22	709.06	1.91	71.25
78	SPI	49	697.41	709.25	1.91	71.27

Figure 3E, F illustrate the direct decomposition of the $P(r)$ function calculated from the atomic coordinates of the PDB model. Atomic pairs were classified into three structural categories: base-base, backbone-backbone, and base-backbone, based on standard nucleic-acid atom definitions. Separate histograms were accumulated for each class using the same bin edges, and their sum exactly reproduces the total curve in the upper panel. No smoothing, scaling, or offset was applied. Peak positions correspond to maxima detected on the unsmoothed total $P(r)$ curve using the same prominence and separation thresholds as in Figure 3C, D.

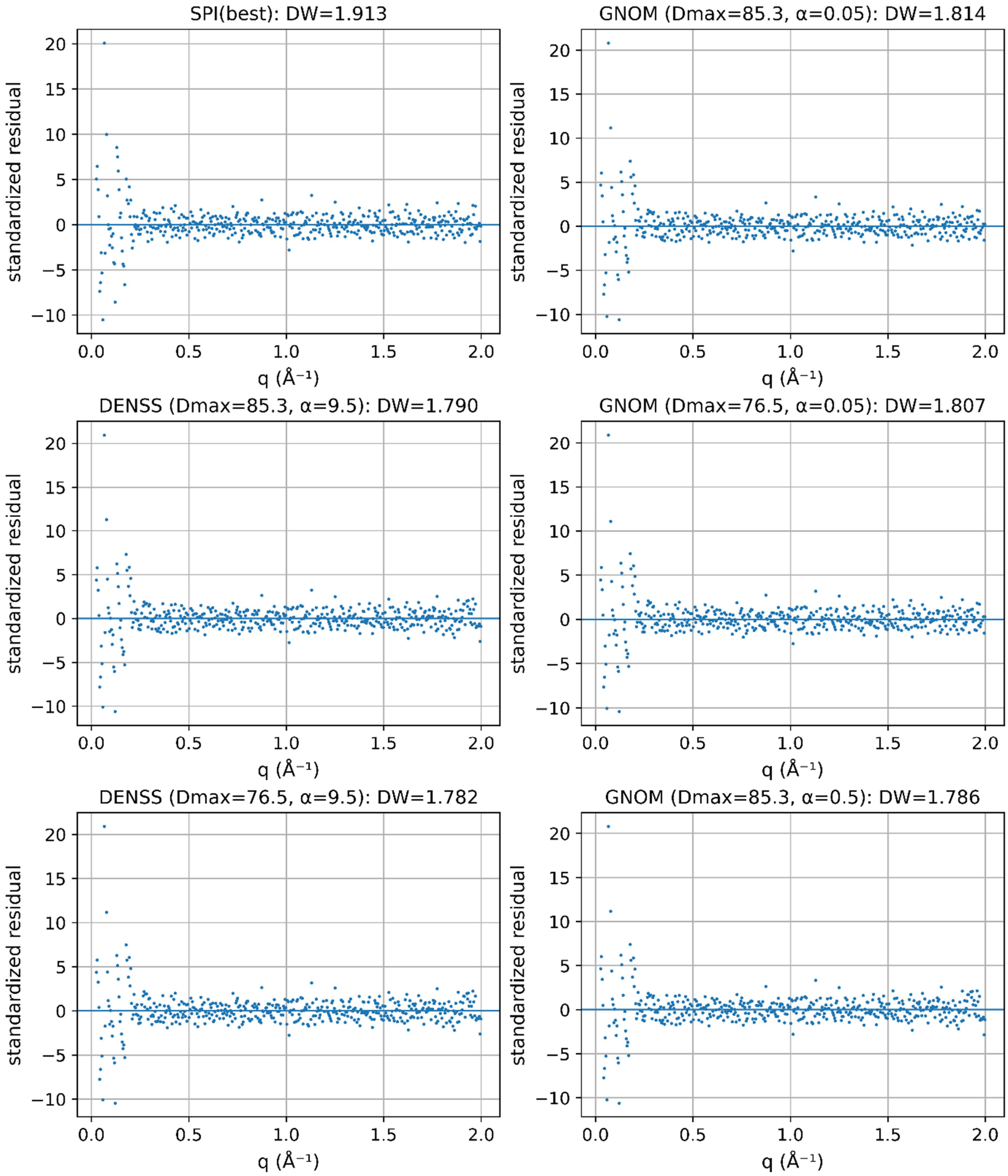

Figure S4 Standardized residuals $(I_{obs} - I_{fit})/\sigma$ for the best SPI and the selected GNOM and DENSS fits. Horizontal line marks zero. Reported Durbin-Watson (DW) statistics are shown in the titles. Residuals are structureless above q = 0.25 Å^{-}, supporting that there are negligible correlations.

S7. $P(r)$ reconstruction with varying q_{max} cut-offs

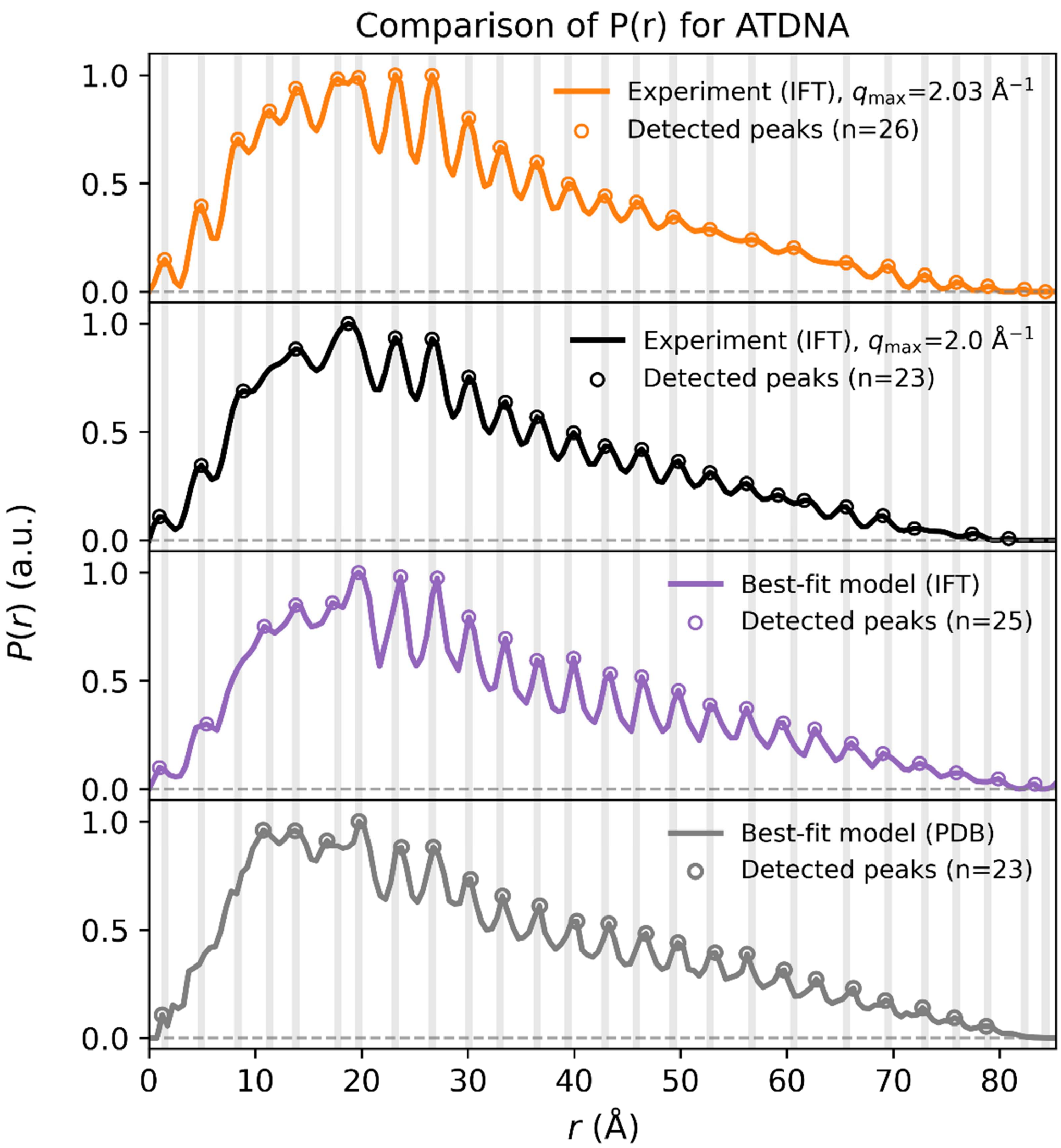

Figure S5 Comparison of $P(r)$ functions reconstructed from the experimental data using q_{max} = 2.03 Å⁻ (orange) and q_{max} = 2.0 Å⁻ (black), alongside the IFT-reconstructed WAXSiS curve (purple) and the model-derived $P(r)$ computed directly from atomic coordinates (grey). Vertical grey bands indicate prominent peaks identified in the experimental $P(r)$ at q_{max} = 2.03 Å⁻ for reference. The emergence of a distinct double peak near 20 **Å** in the orange curve is reproduced more clearly in both the simulated and model-derived profiles. The ability of the calculated curve to resolve this twin feature even at q_{max} = 2.0 Å⁻ likely stems from its sharper high-q scattering, which reflects a single, well-defined structure, whereas the experimental profile represents an ensemble average over multiple conformations in solution and therefore exhibits greater conformational broadening.

S8. Ensemble comparison of experimental and PDB-derived $P(r)$ features

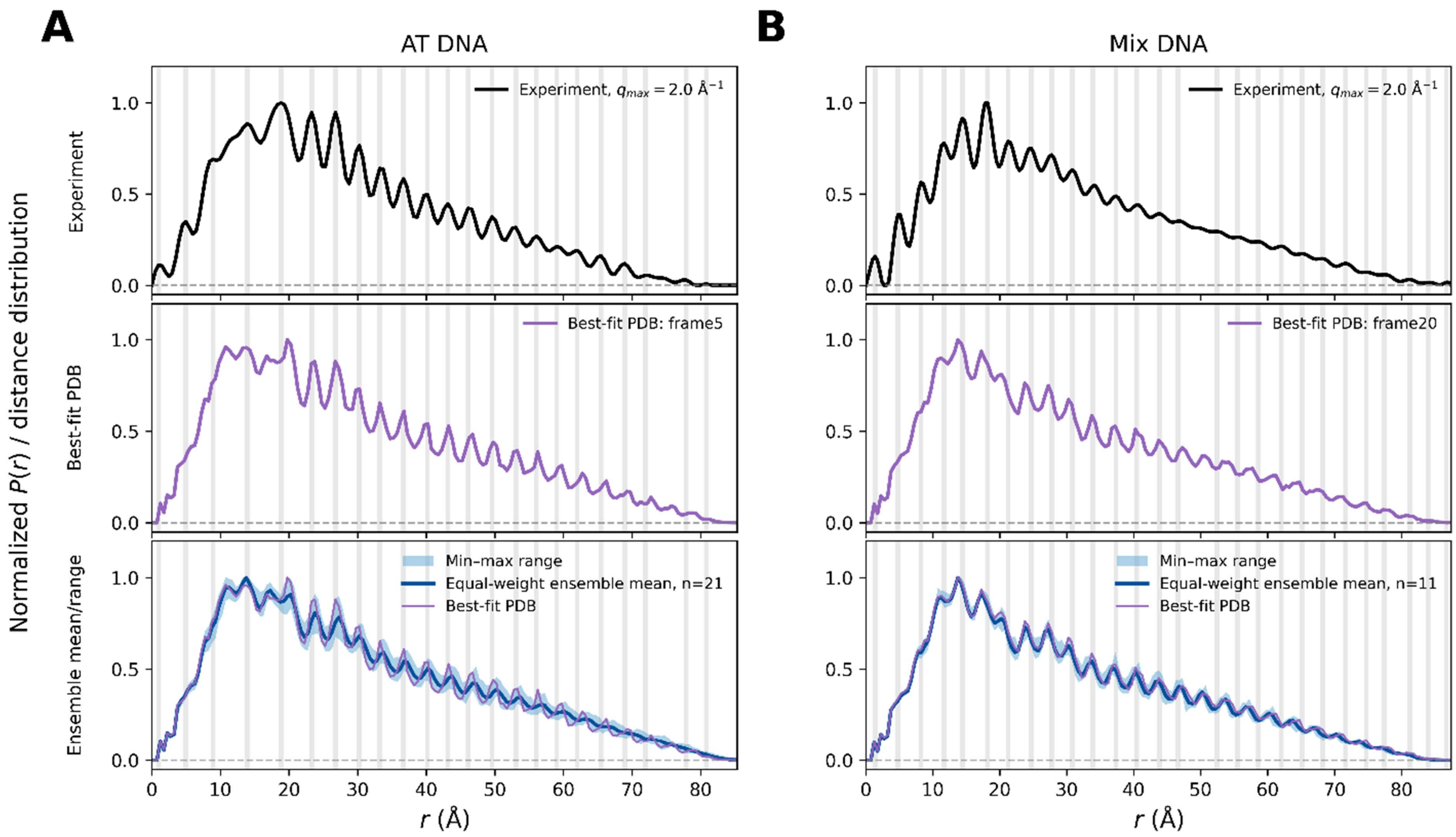

Figure S6 Ensemble comparison of experimental and PDB-derived $P(r)$ features. Experimental $P(r)$ profiles reconstructed from the q_{max} = 2.0 Å⁻¹ SWAXS curves were compared with PDB-derived distance distributions from representative MD structures and structural ensembles. (A) AT DNA. (B) Mix DNA. In each column, the top panel shows the experimental $P(r)$, the second panel shows the PDB-derived distance distribution for the best-fit representative structure, and the third panel shows the equal-weight ensemble average with the shaded region indicating the min–max range across evaluated frames. The best-fit frame is overlaid in the ensemble panel to show its contribution to the ensemble. Grey bands mark peak positions detected from the experimental q_{max} = 2.0 Å⁻¹ $P(r)$. The best-fit PDB-derived distributions retain sharper peak modulation and peak positions that more closely follow the experimental features, whereas the ensemble mean is smoother and shows reduced peak sharpness, consistent with frame-to-frame structural heterogeneity in the MD-derived structures. Scattering profiles computed from the frames used for comparison most closely resembled the measurement and are intended to be representative structural models rather than unique structural solutions.
